# Cobra bite complicated by soft tissue infection with *Salmonella enterica* subsp. *diarizonae.* A case report

**DOI:** 10.1016/j.idcr.2026.e02779

**Published:** 2026-09-23

**Authors:** Benedikte Grønbæk Skov, Marianne Toft, Lars Lemming

**Affiliations:** a Department of Clinical Microbiology, Aarhus University Hospital, Aarhus, Denmark; b Department of Orthopaedic Surgery, Viborg Regional Hospital, Denmark

**Keywords:** *Salmonella enterica* subsp. *diarizonae*, Snakebite, Zoonotic infection, Bacteria, Surgical debridement, Case report

## Abstract

Appropriate first aid and subsequent clinical management are essential to reduce the mortality and morbidity associated with snakebite envenoming. Evidence regarding secondary bacterial infection following venomous snakebites remains limited. We describe the clinical course and microbiological findings in a Danish tourist, who developed a soft tissue necrosis with infection following a venomous cobra snakebite sustained in South Africa. The case describes a secondary soft tissue infection with a polymicrobial culture including *Salmonella enterica* subsp. *diarizonae*. Extensive surgical management, including aggressive debridement, resulted in a favourable clinical outcome despite substantial soft tissue necrosis. We describe how the surgical debridement was managed in Denmark and highlight its importance for a successful outcome.

## Introduction

Snakebite envenoming results in approximately 100,000 deaths annually worldwide. Additionally, around 300,000 individuals suffer amputations or other permanent disabilities [1]. Snakebite envenoming is included in the World Health Organization’s (WHO) list of neglected tropical diseases, with the goal of reducing mortality and disability 50% before year 2030. The highest incidence of venomous snakebites occurs in low- and middle-income countries, where healthcare resources are limited, primarily in Africa, Asia, and South America [1,2].

During a snakebite venom is injected either subcutaneously or intramuscularly. The composition of snake venom varies by species, and the acute toxic effects depend on this composition [3,4]. Appropriate first-aid measures for venomous snakebites are described in the literature and include immobilization of the affected limb to prevent systemic spread via the lymphatic system. However, even when specific antivenom is available, systemic envenoming may be challenging to manage [5,6].

Evidence regarding the management of subsequent infectious complications following venomous snakebite is limited. Necrotic tissue might be infected and serve as a source of bacteraemia and systemic infection, often necessitating systemic antibiotic therapy and surgical debridement [4,5]. Limited data exists regarding frequency of secondary infection following snakebite and the implicated microorganisms. Untreated conditions can lead to life threatening sepsis, chronic infection as well as significant cosmetic and functional impairment [[1], [2], [3], [4]].

## Case presentation

A previously healthy 62-year-old Danish man was bitten by a snake while sitting in a safari vehicle in Kruger National Park, South Africa. The snake had been hiding in the vehicle. The bite was located on the medial side of his left upper arm. He was immediately admitted to a local hospital in a state of shock after 8 h transport, received intensive care, and was administered antivenom 22 h after the bite. Based on the description of the snake, the clinical course and the positive response to antivenom, the clinical presentation was considered most consistent with envenoming by *Naja mossambica*. He was subsequently transferred with sepsis to the intensive care unit in Johannesburg for further treatment. The clinical condition and organ function improved over six days, and he was discharged without planned follow-up. He decided to curtail his holiday and return to Denmark by commercial flight accompanied by his wife.

On day 8 after the snakebite, immediately following his return, he was hospitalized with pain and swelling in the left axilla. After arrival he was afebrile, with a CRP of 26.7 mg/L and leukocyte count of 12.3 × 10^9 cells/L. Clinical examination anteromedially on the left upper arm found marked inflammation around a punctate skin defect with purulent discharge. Ultrasound examination revealed extensive superficial cellulitis near vascular and neural structures. Surgical debridement was performed under general anaesthesia. Intraoperatively, abscess formation and widespread subcutaneous necrosis measuring approximately 25 × 10 cm were observed, extending from the mid upper arm and beneath the pectoralis major along the course of the lymphatic system. The fascia overlying the muscle compartments was intact and viable. Necrotic tissue was surprisingly extensive, and it was excised to healthy margins, and tissue samples were obtained for culture and susceptibility testing. Oral amoxicillin–clavulanate therapy, initiated in South Africa, was continued. The clinical presentation prior to initial surgical debridement is illustrated in Fig. 1. The image is published with the patient’s informed consent.

**Fig. 1:**
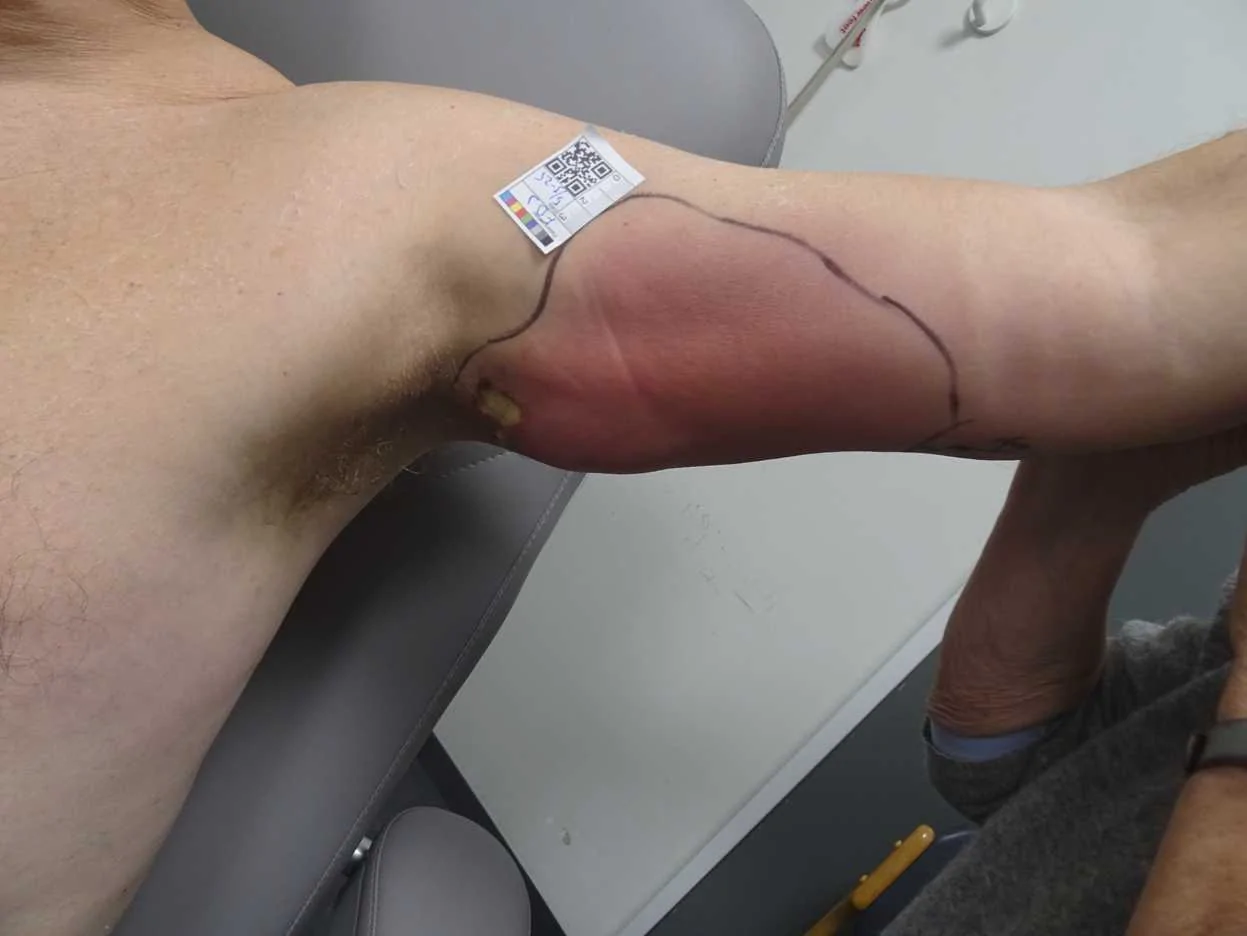

On day 9 a second-look procedure was performed under general anaesthesia, with no further debridement required, and vacuum-assisted closure was applied. On day 11, wound inspection under general anaesthesia revealed a healthy granulating wound bed, with no evidence of necrosis or infection. Following thorough irrigation, the cavity was filled with a Gentacoll® sponge. The wound was closed in layers using deep sutures to eliminate dead space. The patient was subsequently discharged for outpatient follow-up and rehabilitation

*Salmonella enterica* subsp*. diarizonae* was isolated from all tissue specimens obtained during primary surgery, as well as from a swab of the bite wound. The organism was identified by MALDI-TOF MS in polymicrobial cultures that also included *Morganella morganii*, *Enterococcus faecalis*, and *Bacteroides fragilis.* Antibiotic susceptibility testing was performed according to EUCAST breakpoints (version 15.0, 2025/2026) [7], and minimum inhibitory concentrations (MICs) were determined using Etest (BioMérieux, Marcy l’Etoile, France). The *Salmonella enterica* subsp. *diarizonae* was reported susceptible to Ciprofloxacin and Ceftriaxone and was also tested susceptible to Ampicillin. The patient clinically improved during treatment; therefore, the antimicrobial regimen was not modified. Antibiotic therapy was completed as planned after a total treatment duration of 3 weeks. The *Salmonella enterica* subsp. *diarizonae* isolate was subsequently referred to Statens Serum Institut, the Danish laboratory for typing and sequencing. The *Salmonella* genome was particularly large, and detailed genomic characteristics are described in Jørgensen et al., 2026 (submitted). Briefly, the genome size was 6.129 Mb including three large plasmids. Enterobase assigned ST= 13773 and the serotype was phenotypically determined to be IIIb 38:l,z13:z35 using the slide agglutination method. This type is not represented in the White-Kauffmann-Le Minor scheme.

On day 28 the scar had healed satisfactorily. There was a slight reduction in shoulder range of motion due to mechanical skin tightness and early contracture formation. Full range of motion was later regained, and the patient experienced no residual functional impairment.

## Discussion

A high prevalence of snakebite-associated infection has been described, but few studies describe the identification of the possible bacteria present in snakebite wounds in Africa [4]. Snake venom metalloproteases, hyaluronidase, and myotoxic phospholipase A2 contribute to tissue necrosis and degradation of the extracellular matrix [8]. Delay in administration of antivenom will lead to more extensive necrosis. Ideally antivenom is administrated within four hours but is considered effective within 24 h of the snakebite. The composition of its venom may influence the expected efficacy of antivenom, and antivenom administration might carry a risk of allergic reactions, particularly if the antivenom is of equine origin [9]. The 22-hour delay in antivenom administration in this case due to uncertainty regarding the snake species.

We report a rare case of secondary deep wound infection following a cobra snakebite and culture of *Salmonella enterica* subsp. *diarizonae*. Wagener et al. reported that the most common microorganisms identified in snakebite wounds in South Africa were *Enterobacterales* including *Salmonella enterica* and *Enterococcus faecalis* [3], which were also present in this case. *Salmonella enterica* subsp. *diarizonae* is a rare cause of human infection, typically associated with exposure to reptiles such as snakes kept as pets [10,11]. The infection was polymicrobial, but *Salmonella enterica* subsp. *diarizonae* was the only microorganism represented in all diagnostic samples. The bacterial pathogens isolated, particularly *S. enterica* subsp. *diarizonae*, strongly suggest, that the bacteria were inoculated during the snakebite and subsequently established infection in the devitalised and necrotic tissue. Patient history regarding contact with snakes is often obtained only after *Salmonella enterica* subsp. *diarizonae* is identified in clinical specimens [12,13]. In this case though exposure to a snake was evident.

Amoxicillin-clavulanate treatment had already been initiated, and as the patient showed clinical improvement post-operatively, no changes to antibiotic therapy were made. In Denmark, complicated infections with *Salmonella* species are generally treated with ceftriaxone or ciprofloxacin, guided by susceptibility testing.

The commercial flight to Denmark was not associated with any known complications, including hypobaric hypoxia, tissue barotrauma, or thromboembolic events, although recent hospitalisation and immobility may have increased the risk of thromboembolism. Surgical debridement of necrotic tissue is the most critical component of treatment, as it is essential for antibiotic efficacy, the host immune defence, and overall clinical outcome. Large wounds can significantly impair quality of life due to frequent medical visits, immobilisation, recurring infection and potential malodour. Therefore, thorough primary debridement is crucial, with planned secondary evaluation [14]. In this case, the extent of subcutaneous necrosis was surprisingly extensive therefore meticulous debridement was potentially mutilating but undoubtedly necessary.

## CRediT authorship contribution statement

**Benedikte Grønbæk Skov:** Writing – original draft, Project administration, Investigation, Writing – review & editing. **Marianne Toft:** Writing – review & editing. **Lars Lemming:** Writing – review & editing, Supervision.

## Ethics

This case report was in accordance with the declaration of Helsinki principles. There are no names or identifiers throughout this case report.

## Consent

Consent to publish this case and use of health data was obtained by the patient. Informed consent was obtained verbally and in writing by a consent form.

## Funding

The authors did not receive support from any organization for the submitted work.

## Declaration of Competing Interest

The authors declare that they have no known competing financial interests or personal relationships that could have appeared to influence the work reported in this paper.

## Data Availability

No datasets were generated or analyzed during the current study.

## References

[1] World Health Organization. *Snakebite envenoming;* 12 September 2023. Available from: https://www.who.int/news-room/fact-sheets/detail/snakebite-envenoming.

[2] Afroz A., Siddiquea B.N., Chowdhury H.A., Jackson T.N., Watt A.D. (2024). Snakebite envenoming: a systematic review and meta-analysis of global morbidity and mortality. PLoS Negl Trop Dis.

[3] Wagener M., Naidoo M., Aldous C. (2017). Wound infection secondary to snakebite. S Afr Med J.

[4] Bonilla-Aldana D.K., Bonilla-Aldana J.L., Ulloque-Badaracco J.R., Al-Kassab-Córdova A., Hernandez-Bustamante E.A., Alarcon-Braga E.A. (2024). Snakebite-associated infections: a systematic review and meta-analysis. Am J Trop Med Hyg.

[5] Gutiérrez J.M., Calvete J.J., Habib A.G., Harrison R.A., Williams D.J., Warrell D.A. (2017). Snakebite envenoming. Nat Rev Dis Prim.

[6] Goldfrank L.R., Flomenbaum N.E., Nelson L.S., Howland M.A., Lewin N.A., Smith S.W., Goldfrank L.R., Hoffman R.S. (2019). Goldfrank’s Toxicologic Emergencies.

[7] EUCAST. *Breakpoint tables for interpretation of MICs and zone diameters*. Version 15.0, 2025. European Committee on Antimicrobial Susceptibility Testing. Available from: https://www.eucast.org.

[8] Santhosh M.S., Hemshekhar M., Sunitha K., Thushara R.M., Jnaneshwari S., Kemparaju K. (2013). Snake venom induced local toxicities: plant secondary metabolites as an auxiliary therapy. Mini Rev Med Chem.

[9] Juckett G., Hancox J.G. (2002). Venomous snakebites in the United States: management review and update. Am Fam Physician.

[10] Schröter M., Roggentin P., Hofmann J., Speicher A., Laufs R., Mack D. (2004). Pet snakes as a reservoir for Salmonella enterica subsp. diarizonae (Serogroup IIIb): a prospective study.. Appl Environ Microbiol.

[11] Zhou M., Shi Q., Zhang X., Mei L., Ye Y., Fang C. (2021). Salmonella enterica subsp. diarizonae Harboring ST233, ST1263, and ST1845 in Children. Front Cell Infect Microbiol.

[12] Horvath L., Kraft M., Fostiropoulos K., Falkowski A., Tarr P.E. (2016). Salmonella enterica subsp. diarizonae maxillary sinusitis in a snake handler: first report. Open Forum Infect Dis.

[13] Clancy M.M., Davis M., Valitutto M.T., Nelson K., Sykes J.M. (2016). 4th. *Salmonella infection and carriage in reptiles in a zoological collection*. J Am Vet Med Assoc.

[14] Stevens D.L., Bisno A.L., Chambers H.F., Dellinger E.P., Goldstein E.J., Gorbach S.L., Infectious Diseases Society of America (2014). Practice guidelines for the diagnosis and management of skin and soft tissue infections: 2014 update by the Infectious Diseases Society of America. Clin Infect Dis.

